# Regulating the expression of exercise-induced micro-RNAs and long non-coding RNAs: implications for controlling cardiovascular diseases and heart failure

**DOI:** 10.3389/fmolb.2025.1587124

**Published:** 2025-05-20

**Authors:** Guobiao Yang, Wanying Yang

**Affiliations:** ^1^ Department of Physical Education, Xidian University, Xi’an, Shaanxi, China; ^2^ School of Marxism, Xi’an Jiaotong University, Xi’an, Shaanxi, China

**Keywords:** exercise, microRNAs, long non-coding RNAs, cardiovascular diseases, heart failure

## Abstract

The intricate interplay between physical training and non-coding RNAs, specifically microRNAs (miRNAs) and long non-coding RNAs (lncRNAs), has attracted considerable attention in understanding physiological adaptations and pathological conditions. Both miRNAs and lncRNAs are essential modulators of gene expression, influencing various cellular processes, including those related to muscle metabolism, inflammation, and recovery from injury. This review investigates the bifunctional role of miRNAs and lncRNAs in response to physical training, highlighting their involvement in muscle hypertrophy, endurance adaptations, and the modulation of inflammatory pathways. Additionally, we examine how pathological conditions, such as cardiovascular disease, heart failure, can alter the expression profiles of miRNAs and lncRNAs, potentially disrupting the beneficial effects of physical training. The crosstalk between these non-coding RNAs under physiological and pathological states underscores their potential as biomarkers for assessing training responses and therapeutic targets for enhancing recovery and performance. Understanding these interactions may pave the way for novel interventions to optimize health outcomes through tailored physical training programs.

## 1 Introduction

It is widely acknowledged that physical activity has numerous beneficial impacts on health, particularly in preventing and managing long-term conditions such as cancer, metabolic disorders, neurological diseases, musculoskeletal disorders, and cardiovascular disease (Dhuli et al., 2022). This is because physical activity is essential for overall wellbeing. Muscle regeneration depends on satellite cells, diminutive adult stem cells proficient in re-entering the cell cycle after injury or growth stimuli. These cells migrate and proliferate extensively for muscle restoration. The destiny of satellite cells is governed by both intrinsic and extrinsic factors, which may be compromised by ageing, genetic myopathy, and significant muscle atrophy. Surgery is frequently employed in clinical practice; however, its substantial expense and significant dissatisfaction may deter certain patients. Tissue engineering (TE) is currently regarded as an innovative approach for the management of myopathies and the enhancement of regeneration (Ganassi et al., 2022). In addition to causing significant disruptions in various organs and tissues, physical training also causes disturbances in the homeostasis of the body as a whole. The contraction of myofibers is the first step in the movement process, which leads to metabolic and morphological changes in skeletal muscle. Enhanced fuel and oxygen are supplied to the body by the cardiovascular, respiratory, neural, and endocrine systems responsible for supporting activity (Plaza-Diaz et al., 2022; Qiu et al., 2023; Chen et al., 2022).

Postnatal development of skeletal muscle in humans primarily results from the hypertrophy of preexisting muscle fibers, rather than from cellular proliferation. Historical evidence of muscle fiber division or bifurcation originates from the 19th century, and contemporary research corroborates this with findings from regenerating and stress-induced muscle fibres. Nonetheless, there exists circumstantial evidence of hypertrophy and hyperplasia induced by exercise, primarily derived from subsequent observations of variations in fibre size or quantity. Researchers have created three animal models to replicate exercise-induced muscle hypertrophy in humans (Snijders et al., 2015). The ageing population poses a global challenge to governments, as age-associated ailments such as sarcopenia become more widespread. Sarcopenia is a clinical syndrome marked by the age-related decline in skeletal muscle mass, strength, and function, frequently observed in elderly patients with chronic illnesses. Alterations in lean mass are pivotal factors influencing the progression of cardiovascular disease. Sarcopenia may lead to impaired physical function and diminished cardiopulmonary function in elderly patients with cardiovascular diseases via shared pathogenic mechanisms. Furthermore, cardiac modifications in the left ventricle, especially among elderly individuals with skeletal muscle sarcopenia, have been thoroughly investigated for decades (He et al., 2021). There is an increasing interest in comprehending the myocardial alterations that accompany these changes, resulting in a revitalised emphasis on the cardiac-skeletal muscle axis. Interestingly, sarcopenia, a condition marked by age-associated muscle deterioration, is also linked to Type 2 Diabetes Mellitus (T2DM) and dementia. It elevates Insulin resistance and the susceptibility to type 2 diabetes mellitus, resulting in cognitive deterioration and an increased risk of dementia. Sarcopenia may serve as a mediator between these conditions, intensifying cognitive decline in elderly individuals. Recent studies have highlighted the correlation between sarcopenia and dementia, rendering it a critical area of research. Comprehending these associations can facilitate the formulation of strategies to promote healthy ageing and avert dementia in elderly individuals with T2DM. Further investigation is necessary to improve understanding of these complex relationships (Liu et al., 2024).

**FIGURE 1:**
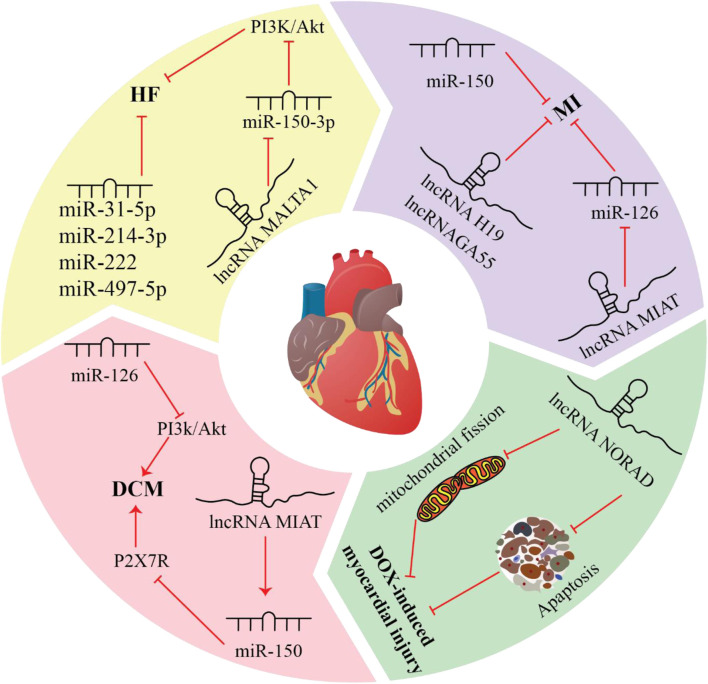
Exercise-induced miRNAs and lncRNAs are crucial in the progression of various cardiovascular diseases, including hypertension, dilated cardiomyopathy, atherosclerotic vascular diseases, myocardial ischemia-reperfusion injury, myocardial infarction, heart failure, and doxorubicin-induced cardiomyopathy.

It has been established that non-coding RNAs, also known as ncRNAs, have the potential to act as mediators in the physiological processes that are associated with exercise adaptation. In addition to small nucleolar RNA, ribosomal RNA, circular RNA, transfer RNA, long non-coding RNA, and microRNA, the comprehensive catalog of non-coding RNAs also includes many other types of RNA (Wadley et al., 1985; Piergentili and Sechi, 2024). Throughout the 1950s, the first noncoding RNAs were found. lncRNAs, miRNAs, small interfering RNAs (siRNAs), and PIWI-interacting RNAs (piRNAs) were added to the repertoire of recognized non-coding RNAs as a result of this research. Additionally, the research identified multiple RNA categories, including snoRNAs that are involved in the processing and splicing of rRNA (Burgos et al., 2021; Chen and Kim, 2024). In light of the rapidly growing amount of data, this study investigates the synthesis and function of microRNAs (miRNAs) and long non-coding RNAs (lncRNAs) in skeletal muscle. Moreover, Physiological enhancements resulting from exercise appear to influence the expression of diverse miRNAs, encompassing myo-miRNAs, c-miRNAs, and lncRNAs. Consequently, the examination of exercise-induced modifications in miRNAs and lncRNAs expression in cardiovascular disease and heart failure may yield novel insights into the epigenetic modifications prompted by exercise. However, MiRNAs and lncRNAs may contribute to health-related enhancements; however, their molecular mechanisms are inadequately comprehended in individuals with cardiovascular disease and heart failure. Prior studies have concentrated on identifying myo-c-miRNAs or lncRNAs that exhibit differential expression in reaction to both acute and chronic physical exercise in human. Furthermore, investigations concentrated on enhancing health outcomes for patients, especially those impacted by cardiovascular disease and heart failure (Afzal et al., 2024). This review aims to examine the impact of exercise on the expression of myo- or c-miRNAs and lncRNAs in cardiovascular disease and heart failure, highlighting potential molecular pathways for improved health outcomes (Figure 1).

## 2 Biogenesis and mechanism of action ncRNAs

MicroRNAs, small and plentiful molecules with seed sequences, facilitate target recognition, improving interaction with miRNA processing machinery for varied identification and prediction (Desvignes et al., 2015). MicroRNAs are essential in modulating adaptive processes, including muscle atrophy, cardiovascular disease, and aging (O'Brien et al., 2018; Seo et al., 2024). Recent studies indicate that lncRNAs are critical to regulating locus-specific gene expression by functioning as cofactors, competitors, or decoys for RNA-binding proteins and microRNAs (Zhang et al., 2019).

**FIGURE 2:**
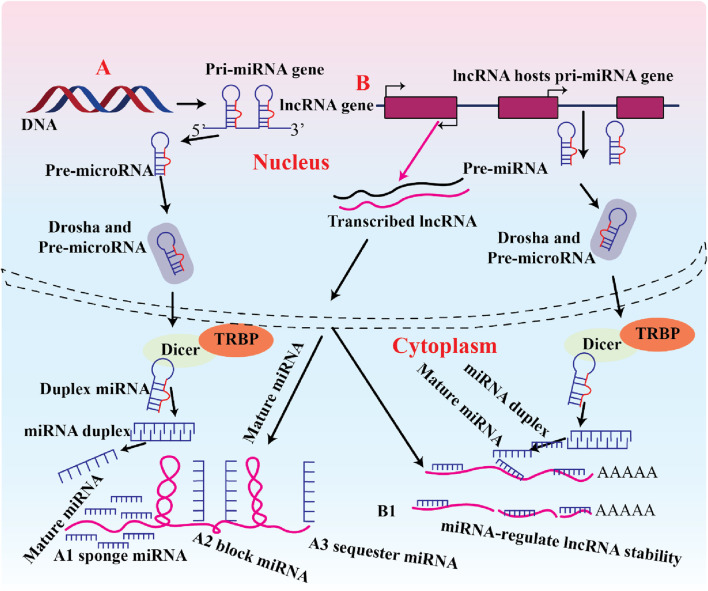
Depicts the biogenesis of lncRNA-miRNA interactions, encompassing binding, blocking, sequestering, and processing of lncRNA genes, wherein miRNAs destabilise lncRNAs and modulate exercise.

Other ncRNAs subtypes include cirRNA, siRNA, tiRNA, and piRNA.Circular RNAs, which are non-coding RNAs, form closed loops in mammals and regulate gene expression via miRNA sponges or RNA-binding proteins, thus influencing gene transcription (Greene et al., 2017). Small interfering RNAs, encompassing natural antisense, trans-acting, and heterochromatic siRNAs, impede protein synthesis by cleaving target mRNA at a designated site within the siRNA-RISC complex (Deng et al., 2018). Transfer RNA-derived small RNAs (tsRNAs), a novel class of regulatory non-coding RNAs, range from 15 to 42 nucleotides in length and fulfill diverse biological functions through protein interactions, translation inhibition, and gene expression regulation (Zong et al., 2021). PiRNAs, found in germline DNA, inhibit transposable elements. PIWI proteins, integrated into Argonaute proteins, cleave target mRNAs while maintaining the integrity of germline DNA (Iwasaki et al., 2015) (Figure 2).

## 3 The role of microRNAs and lncRNAs in the exercise and in skeletal muscle adaptation to exercise

MicroRNAs in skeletal muscle, cardiac tissue, and vasculature have been the subject of recent research that has shed light on their role in exercise adaptations and brought to light potential therapeutic targets (Güller and Russell, 2010). The regulatory roles that microRNAs play in physical fitness will be investigated in this review, which will focus on these domains (Wang et al., 2018). Despite the limited characterization that has been done, functional lncRNAs fulfill the role of essential regulatory molecules in cells. They have piqued people’s interest in their possible role in both health and disease through their discovery. Another putative is responsible for encoding myoregulin (MLN), a micropeptide that is specific to skeletal muscle and was recently discovered. Integration of MLN into the SR membrane, colocalization of MLN with SERCA1, and modulation of Ca2+ management through inhibition of SERCA pump activity were all observed. The exercise performance of MLN-KO mice was significantly improved, as was their ability to handle calcium in muscle tissue (Moore and Uchida, 2020; Anderson et al., 2015). Although the study discovered a correlation between lncRNAs and skeletal muscle during physical training, Further research is necessary to fully understand the role of lncRNAs in the exercise process. A recent study indicates that lncRNAs may inhibit osteoporosis and demonstrates that exercise modulates the expression levels of particular lncRNAs in osteoporotic mice. Within the context of this process, it is conceivable that specific lncRNAs, such as lncRNA H19 and LOC102637959, are involved.

The adaptation of skeletal muscle to exercise is affected by the structure of contractile proteins, mitochondrial functionality, metabolic regulation, intracellular signaling, and transcriptional responses such as MEF2, HDACs, and NRFs (Smith et al., 2023). Some physiological changes take place in myofibers, which are the primary components of muscle tissue, after exercise to ensure that they continue to function at their highest potential. There have been experimental studies that have demonstrated fitness-related changes in microRNAs. These microRNAs are thought to influence muscle myogenesis, muscle mass, and metabolism in skeletal muscle by modulating specific genes that are involved in myogenic regulatory signaling (Ultimo et al., 2018). The dysregulation of microRNAs in skeletal muscle following physical training brings to light the connection between exercise-induced physiological changes and changes in muscle function. Comprehending miRNA biogenesis in skeletal muscle following exercise could enhance the efficacy of therapies. MicroRNAs are essential in muscle function during physical training (Zacharewicz et al., 2013) (Table 1).

**TABLE 1:** The cellular impact of the major microRNAs involved in the exercise.

microRNA	Exercise type	Origin/Expression	Cellular effect	References
miR-21	Acute exercise training	Serum/increased	Promoting angiogenesis, anti-inflammatory, and anti-apoptosis effects	Zhou et al. (2020)
miR-146a	Acute exercise training	Plasma/decreased	The expression of CD80 and glucose transporter 3, essential for the inflammatory response, is modified and diminished during acute exercise	Balchin et al. (2023)
miR-221	Acute exercise training	Serum/increased	The preliminary pro-inflammatory mechanisms that arise during acute exercise, particularly at elevated intensities, are a crucial element in the body’s stress response	Ma et al. (2015a)
miR-1	Endurance training	Soleus muscle/decreased	modulating muscle growth and differentiation by regulating SRF and MEF2 activity	Bahrami et al. (2023)
miR-133a/b	Endurance training	Soleus muscle/increased	modulating muscle growth and differentiation by regulating SRF and MEF2 activity	Bahrami et al. (2023)
miR-206	Endurance training	Skeletal muscle/increased	Involved in the function of myostatin in skeletal muscle development, growth, adaptation, regeneration, and muscle-related disorders, with the objective of enhancing muscle strength	Ma et al. (2015)
miR-29a/b/c	high-intensity intermittent exercise training	Skeletal muscle/increased	Regulation of VEGFA, COL4A1, and COL4A2 expression through the PI3K/Akt signaling pathway to enhance the proliferation and migration of vascular endothelial cells and skeletal muscle angiogenesis	Chen et al. (2020)
miR-370-3p	resistance training	Skeletal muscle/decreased	exosomal miR-370-3p aggravated I/R-induced BBB disruption by targeting MPK1	Gu et al. (2023)
miR-211	Wheel Running	Skeletal muscle/decreased	Modulates Feeding Behavior and is Involved in Inflammation Processes in the ARC	Rapps et al. (2024)
miR-205-5p	Moderate physical exercise and strength training	Skeletal muscle/decreased	Inducing the inflammatory response in Allergic rhinitis (AR) by targeting BCL6, which may be a potential therapeutic target for AR	Zhang et al. (2021)
miRs-199a-3p and −19b-3p	endurance training (MICT) and high-intensity interval training (HIIT)	Skeletal muscle//increased	independently increases NO bioavailability by promoting eNOS activity and reducing its degradation, thereby supporting VEGF-induced endothelial tubulogenesis and modulating vessel contractile tone	Joris et al. (2018)
miR-27a	Swimming	Skeletal muscle/increased	The miR-23a/27a-24–2 cluster is implicated in chronic kidney disease-induced muscle atrophy in murine models, implying that physical activity is essential for preserving muscle mass	Wang et al. (2017a)
miR-155	Regular physical activity	Skeletal muscle/increased	the primary inflammatory response following muscle injury can be mitigated by obstructing the negative regulator of the JAK-STAT signaling pathway	Nie et al. (2016b)
miR-181a	Aerobic	Skeletal muscle/increased	The regulation of the oxidative stress response has been examined concerning myocardial function	Borja-Gonzalez et al. (2020)
miR-24-2	Aerobic	Skeletal muscle/increased	This encompasses the regulation of apoptosis, immune function, protein-membrane trafficking, and transcription	Balchin et al. (2023)

Skeletal muscle can undergo metabolic and functional adaptations as a result of the activation of signaling pathways from physical exercise. In this response, PGC-1α plays a crucial role as a mediator, contributing to the regulation of angiogenesis, inflammation, mitochondrial metabolism, and β-oxidation, respectively (Olesen et al., 2010). In male C57Bl/6J mice, endurance exercise resulted in a significant increase in the expression of miR-107, -181, and -1 (Safdar et al., 2009). The research indicates that microRNAs significantly regulate mitochondrial biogenesis in skeletal muscle following physical activity, suggesting that post-transcriptional regulation of PGC-1α by miR-23 could play a role in adaptation in skeletal muscle, highlighting the importance of microRNAs in skeletal muscle function. Specifically, the study reveals that exercise has a significant effect on miR-696, which targets PGC-1α. In murine myoblast C2C12 cells, the study discovered that the expression of miR-494 decreased during the process of myogenic differentiation (Aoi et al., 2010). It was observed that there was an increase in mitochondrial DNA as well as predicted target genes such as mitochondrial transcription factors A and Foxj3, which are essential for the construction of mitochondria (Kang et al., 2018). Following endurance exercise, the expression of miR-494 in the skeletal muscle of C57BL/6J mice decreased, indicating that it plays an important role in mitochondrial biogenesis through the mechanisms of mtTFA and Foxj3. Analysis was performed on the regulation of mRNA components in the miRNA biogenesis pathway (Drosha, Dicer, and Exportin-5), as well as muscle-specific miRNAs (miR-206, -133a, -1, and -133b), and many miRNAs that are altered in muscle myopathies (miR-181, -29, -9, -23, and -31) (Yamamoto et al., 2012; Kirby et al., 2015). After undergoing an acute short-term exercise, it was observed that the miRNA biogenesis pathway, along with miR-133a, -181a -1, and -133b, exhibited an increase. On the other hand, miR-31, -23a -9, and-23b, exhibited a decrease. Following twelve weeks of endurance training, the expression of myomiRs, specifically miR-206, -133b, -1, and -133a, was found to decrease in human muscle biopsies (Russell et al., 2013; Ultimo et al., 2018). MyomiR levels returned to their baseline level fourteen days after regular training had been completed. There is still a lack of knowledge regarding the precise mechanisms that myomiRs use to influence human physiology in response to exercising for long periods. Through the regulation of processes such as gene transcription and mitochondrial biogenesis, exercise-induced microRNA changes facilitate muscle adaptation to exercise. These alterations regulate extremely important processes, including the regeneration of muscle tissue, the transcription of genes, and the biogenesis of mitochondria (Nielsen et al., 2010). Myogenesis and muscle function are governed by diverse signalling pathways, modulated by an array of miRNAs, encompassing both ubiquitous and tissue-specific variants, throughout muscle differentiation and activity. MiR-133a, a constituent of the myomiRs family, is essential for myogenic differentiation, muscle fibre type specification, and mitochondrial biosynthesis in skeletal muscle (Wang, 2013). Dey’s 2012 study (Dey et al., 2012) demonstrated that miR-26a inhibits the expression of Smad1 and Smad4, thereby affirming the role of miRNAs in myogenic differentiation. In addition, MiR-206 and miR-29 can attenuate the inhibitory effects of TGFβ signaling on myogenic differentiation by suppressing Smad3 expression, which subsequently downregulates HDAC4 expression. The myomiRs miR-1 and miR-206 modulate Pax7 expression. An antimiR suppresses the activity of miR-1 and miR-206, thereby enhancing Pax7 expression and cellular proliferation. Pax7 regulates miR-486, a member of the myomiR family that is upregulated during differentiation (Chen et al., 2010). Myostatin, a growth factor-8, modulates skeletal muscle mass by inhibiting the IGF-1/Akt/mTOR pathway, thereby influencing muscle protein synthesis, although the precise mechanism is not fully understood (Rodriguez et al., 2014). The research identified miR-486, a crucial regulator of the IGF-1/Akt pathway, as a target of myostatin signaling. In myostatin knockout mice, miR-486 expression was elevated, and myostatin signaling suppressed its activity. The overexpression of miR-486 induced myotube hypertrophy and maintained skeletal muscle mass (Hitachi et al., 2014). Suppression of MiR-486 diminished Akt activity in C2C12 myotubes, whereas MiR-145 safeguarded cardiomyocytes from reactive oxygen species-induced damage by inhibiting mitochondrial apoptosis, averting Ca2+ overload, and obstructing cardiomyocyte hypertrophy. Reduced miR-145 levels may facilitate the expression of genes associated with muscle atrophy (Cha et al., 2013).

Changes in skeletal muscle associated with aging impact mobility and overall quality of life. The capacity for physical activity is essential for longevity. Older males exhibit elevated levels of pri-miR-1 and -133a, whereas younger males demonstrate heightened miR-1 expression in skeletal muscle (Ultimo et al., 2018; Distefano and Goodpaster, 2018). This suggests that younger males have a different age-related muscular response to fitness than older men. The miR-126-IGF-1 axis is responsible for regulating exercise-induced adaptations in skeletal muscle, which indicates that dysregulation occurs with aging. Certain microRNAs may play a role in the progression of sarcopenia and have the potential to be used as therapeutics for the aging process. Resistance training can stimulate Akt-mTOR signaling, which is decreased in conditions associated with age-related muscle atrophy (Rivas et al., 2014). Analysis of 26 microRNAs that target the Akt-mTOR pathway revealed that dysregulation occurs as a result of both aging and physical activity. The microRNA family known as miR-99/100 is accountable for the regulation of Akt-mTOR signaling and muscle protein synthesis in individuals of all ages, including those who are young and those who are elderly (Gao et al., 2023). According to this, it appears that the modifications of miRNA that are induced by exercise may be able to help mitigate the effects of age-related muscle deterioration and muscle disorders (Table 2).

**TABLE 2:** microRNAs and their Molecular mechanism in muscle disorders.

microRNA	Disease type	Molecular mechanism	References
miR-30a-3p, miR-30e-3p, and miR-199b-5p	Dermatomyositis	A correlation between the downregulation of miR-30a-3p, miR-30e-3p, and miR-199b-5p in diabetes mellitus and the upregulation of target genes triggered by type I interferon	Muñoz-Braceras et al. (2023)
miR-96-5p	Idiopathic inflammatory myopathies (IIMs)	Dysregulation of interferon signaling, anti-viral response, and T-helper cell pathways, and indicates its possible role in the regulation of ADK	Parkes et al. (2020)
miR-26a	Cardiomyopathy and muscle atrophy	Promotes myoblast differentiation, decreased muscular atrophy, and ameliorated cardiomyopathy symptoms, indicating that the augmentation of its expression effectively attenuates insulin resistance in chronic kidney disease	Wang et al. (2019a)
miR-155	Duchenne muscular dystrophy (DMD)	Influences myoblast proliferation and differentiation into myotubes during *in vitro* myogenesis	Lopes et al. (2024)
miR-378	Duchenne muscular dystrophy (DMD)	Modifies carbohydrate and lipid metabolism in dystrophic mdx mice	Podkalicka et al. (2022)
miR-133b	Duchenne muscular dystrophy (DMD)	The mdx mouse model markedly diminishes muscle cross-sectional area, affecting various signaling pathways including apelin, PPARα, and STAT3	Kiełbowski et al. (2024)
miR-206	Duchenne muscular dystrophy (DMD)	Reduces via AAV9-anti-miR-206, improving motor function and mitigating dystrophic pathology, indicating a novel therapeutic strategy for DMD.	Bulaklak et al. (2018)
miR-199a-5p	Duchenne muscular dystrophy (DMD)	Affects multiple regulatory elements within the WNT signalling pathway, including FZD4, JAG1, and WNT2, which govern myogenic cell proliferation and differentiation	Alexander et al. (2013)
miR-146a	Duchenne muscular dystrophy (DMD)	Dystrophin production is inhibited in Becker muscular dystrophy and a murine model of Duchenne muscular dystrophy exon skipping as a result of a muscular dystrophy feedback mechanism	McCormack et al. (2023)
miR-486	Duchenne muscular dystrophy (DMD)	The PTEN/AKT pathway is modulated in dystrophin-deficient muscle and is essential for the regulation of DMD muscle	Alexander et al. (2011)
Alexander et al. (2013) miR-503, miR-322/424	Muscle atrophy and cachexia	Induce myogenesis interfering with the progression through the cell cycle	Wang et al. (2019b)
miR-29b	Muscle atrophy	This substance is a crucial regulator of multiple forms of muscle atrophy, including denervation, dexamethasone treatment, fasting, cancer cachexia, aging, and angiotensin II.	Li et al. (2017)
miR-34a-5p and -miR-449b-5p	Sarcopenia	Significantly increased the expression of a key senescence gene sirtuin 1 (SIRT1) and other genes related to the mitogen-activated protein kinase (MAPK) pathway, which regulates aging processes	Brzeszczyńska et al. (2020)
miR-431	Sarcopenia	Inhibit TGF-β signaling and improve muscle regeneration and myogenic capacity of myoblasts	Lee et al. (2015)
miR-27a	Cancer cachexia	Downregulate myostatin (Mstn), a member of the TGFβ family, responsible for SSC activation and myoblast proliferation	Huang et al. (2012)
miR-199	Cancer cachexia	Linked to Cav1 and transcription factor Jun-B (Junb) regulation. Junb regulates gene expression on multiple levels	Brzeszczyńska et al. (2020)
miR-21	Cancer cachexia	The proposed method entails fusing with muscle cells and activating the toll-like receptor (TLR7/8), subsequently inducing apoptosis	He et al. (2014)
miR-675	Sarcopenia	Modulating the IGF1R/Akt/FoxO pathway could be a viable therapeutic target for addressing skeletal muscle atrophy	Zhang et al. (2023)
miR-146b	Sarcopenia	Regulates the dysfunction of vascular smooth muscle cells via repressing phosphoinositide-3 kinase catalytic subunit gamma	Zhuang et al. (2021)
miR-125b	Sarcopenia	Inhibits the proliferation of vascular smooth muscle cells induced by platelet-derived growth factor-BB	Wang et al. (2021)
let-7	Sarcopenia	Targeting PAX7 and IL-6 in senescent muscle and oculopharyngeal muscular dystrophy (OPMD), a condition analogous to senescent muscle, leads to diminished muscle regeneration and functional decline	Gidaro et al. (2013)
miR-34a	Sarcopenia	Induce senescence in vascular smooth muscle cells and cardiomyocytes and increase cardiac fibrosis	Badi et al. (2015)

MicroRNAs possess the capacity to modulate genes associated with mitochondrial processes, including ATP synthesis, oxidative stress, and overall mitochondrial functionality. MiR-4485 is associated with the regulation of mitochondrial respiratory complex I activity, signifying its role in mitochondrial metabolism. Exercise has been also shown to modulate miR-696, which is associated with PGC-1α, an essential regulator of mitochondrial biogenesis (Sripada et al., 2017). Another study showed the critical function of miR-133a in mitochondrial biogenesis within skeletal muscle, exercise endurance, and adaptation to exercise training (Nie Y. et al., 2016). In addition, Research indicates that miRNAs may indirectly affect the activity of the TOM complex during exercise by modulating genes associated with TOM complex functionality and mitochondrial protein import. For example, miR-494 indirectly affects TOM complex activity by contributing to mitochondrial biogenesis and exercise adaptation (Yamamoto et al., 2012). miR-106b has been identified as a regulator of genes associated with mitochondrial biogenesis and dynamics, potentially influencing the functionality of the TOM complex (Zhang et al., 2013). miR-124a is essential for mitochondrial function, impacting mitophagy and mitochondrial dynamics, which may indirectly influence the TOM complex (Saikia et al., 2023).

The results have improved our comprehension of exercise’s influence on lncRNA expression and offered evidence for the underlying mechanism of this regulation (Chen et al., 2021). The evidence should facilitate the development of novel therapeutic interventions for the prevention and treatment of osteoporosis. Another study demonstrated that the lncRNA CRNDE was highly expressed in exosomes that were derived from long-term exercise. While inhibiting the progression of myocardial infarction, CRNDE knockdown led to an increase in apoptosis and oxidative stress in cardiomyocytes. CRNDE acted as a sponge for miR-489-3p, which is responsible for affecting the expression of Nrf2. The inhibition of miR-489-3p was able to effectively reverse the effects of CRNDE depletion on cardiomyocytes that were working under hypoxia. These discoveries presented a potentially useful therapeutic option for the treatment of myocardial infarction (Han et al., 2024). The research conducted by Wohlwend et al. revealed that the long noncoding RNA CYTOR, which is upregulated in human, rat, and mouse muscle post-exercise, enhances myogenic differentiation and facilitates the formation of type II muscle fibers *in vitro*, underscoring the substantial influence of exercise and aging on skeletal muscle. Manipulation of cytor expression can modify skeletal muscle mass, strength, and performance in both mice and humans. A single-nucleotide polymorphism associated with elevated CYTOR expression in human skeletal muscle and enhanced distance in the Helsinki Birth Cohort Study may facilitate future interventions to improve myogenesis (Wohlwend et al., 2021).

## 4 The role of microRNAs and lncRNAs in the cardiovascular adaptation to exercise and in cardiovascular disease and heart failure

Ischemia-reperfusion injury and myocardial infarction are both protected against by exercise, which also induces physiological cardiac hypertrophy in athletes’ hearts. Exercise also promotes cardiac hypertrophy in athletes, which protects against these injuries. Cardiovascular hypertrophy that is induced by exercise is considered to be protective and has the potential to occasionally improve cardiac function. This is in contrast to pathological hypertrophy, which can lead to a poor prognosis and heart failure (Calvert et al., 2011). As a result of their physiological adaptation, microRNAs have garnered attention in the field of cardiovascular health and physical activity. This is because of their ability to increase heart size, cardiac output, injury resistance, and improve vascularization characteristics. MiRNAs are becoming more widely recognized for their physiological adaptations, which include increased heart size, cardiac output, injury resistance, and improved vascularization (Liu et al., 2017; Nappi et al., 2023). These adaptations contribute to the potential role that miRNAs could play in the realm of physical activity and cardiovascular health.

Studies indicate that miR-1 and -133 are downregulated in rat hearts due to treadmill training and in transgenic mice exhibiting cardiac overexpression of Akt1. MiR-222, -34a, and -210 are downregulated in physiological hypertrophy and upregulated in dominant-negative PI3K transgenic hearts. In rats undergoing 8 weeks of swim training, the cardiac expression of miR-21, -144, and -145 was elevated, whereas miR-124 was diminished. In exercised hearts, PTEN and TSC2 levels were diminished, whereas PI3K(110α) levels were elevated. The data indicate that certain miRNAs modified by exercise influence PI3K/AKT/mTOR signaling, a crucial regulator of the cardiac response to exercise (Fathi et al., 2020). Soci and colleagues identified 87 differentially expressed cardiac miRNAs following 10 weeks of exercise in comparison to sedentary controls. These miRNAs, comprising 48 upregulated and 39 downregulated species, were implicated in pathological and stress-induced cardiac hypertrophy (Soci et al., 2011). Nonetheless, miR-1, -133a, and -133b diminished following exercise training, whereas miR-29c was elevated in the hearts of exercised subjects. In exercised rats, miR-27a and -27b levels increased, whereas miR-143 levels decreased, indicating that specific miRNAs targeting genes of the renin-angiotensin system are dynamically regulated by exercise training and may influence the development of cardiac hypertrophy (Liu et al., 2017). Martinelli et al. (Martinelli et al., 2014) identified 35 differentially expressed miRNAs following 1 week of exercise and 25 differentially expressed after 5 weeks of training, illustrating the temporal regulation of cardiac miRNAs during exercise training.

A study investigating miRNAs in the hearts of mice subjected to rigorous swimming or voluntary wheel running identified 124 differentially expressed miRNAs in the hearts of wheel-running mice and 55 differentially expressed miRNAs in the hearts of swimming mice, relative to sedentary controls (Inukai et al., 2012). The research indicates that forced swim training may elicit a stress response absent in voluntary wheel running. Sixteen miRNAs, including miR-222, were independently confirmed to be consistently regulated in both models (Liu et al., 2015). Ramasamy et al. (2015) conducted a comparable study revealing that more than 80% of the 201 identified miRNAs exhibited differential regulation. The research indicates that these miRNAs are linked to cardiac hypertrophy and apoptosis. Cardiac development and physiological stimuli or pathological stress induce hypertrophic growth, increasing in cardiomyocyte size. Numerous lncRNAs, such as cardiac hypertrophy-associated transcript (Chast) and cardiac hypertrophy-associated epigenetic regulator (Chaer), participate in this process and exhibit differential regulation in reaction to pathological stress. These transcripts are upregulated in mice after transverse aortic constriction (TAC) and downregulated following TAC (Mably and Wang, 2024). Research indicates that cardiac lncRNAs, including H198 and NRON, may function as therapeutic targets in pressure overload induced by TAC. These lncRNAs can confer cardioprotection in mouse models of TAC, either by targeted knockdown or deletion of specific lncRNAs or through overexpression (Busscher et al., 2022). LncRNAs are integral to pathological cardiac hypertrophy, yet their involvement in physiological growth processes remains inadequately understood. Physiological hypertrophy induced by exercise safeguards against cardiac injury and heart failure, and is associated with cardiomyogenesis. The sole lncRNA essential for exercise-induced physiological cardiac hypertrophy in mice is cardiac physiological hypertrophy-associated regulator (CPhar), as evidenced by a forced swim training model. Research indicates that lncExACT1 exhibits specific mechanisms of action, underscoring the distinctive characteristics of lncRNAs (Gao et al., 2021). It is located in the nuclei and cytoplasm of cardiomyocytes, exhibiting a 2.5-fold enrichment in the nucleus. In the cytoplasm, it suppresses the cardiac-expressed miRNA miR-222, which is essential for physiological cardiac growth and exercise-induced cardiomyogenesis. In the nucleus, it associates with the promoter region of the adjacent gene DCHS2 and enhances its transcription. Nuclear lncRNAs modulate gene expression by interacting with the promoter region of the adjacent DCHS2 gene. They can be categorised as locally modified chromatin structures or distally executed functions. lncExACT1 functions locally by binding to the promoter region of the DCHS2 gene, but exogenous expression of the lncExACT1 transcript can mediate this effect. DCHS2 is positively modulated by lncExACT1; overexpression of lncExACT1 in mice leads to the upregulation of DCHS2, while the inhibition of lncExACT1 is associated with the downregulation of DCHS2(102). The parallel expression patterns are consistent in patients with human heart failure, where both lncExACT1 and DCHS2 are upregulated. DCHS2 is essential and adequate for the effects of lncExACT1 on cardiomyocyte growth, indicating it serves as a downstream effector of lncExACT1 activity (Li et al., 2022).

DCHS2 regulates cell proliferation via the Hippo/Yap1 signaling pathway, a conserved mechanism present in various organ systems, including the heart. Exercise and the inhibition of lncExACT1 or DCHS2 augmented the nuclear fraction of Yap1, leading to the expression of downstream Yap1 targets. The knockdown of DCHS2 diminished the cytoplasmic levels of phosphorylated MST1/2, a fundamental regulatory protein in the Hippo pathway. The data indicate that lncExACT1 and DCHS2 are new regulators of cardiac Yap1, playing a role in exercise-induced cardiac hypertrophy and cardiomyogenesis. Nevertheless, previous research indicates that postnatal activation of cardiac Yap1 does not enhance cardiomyocyte size, implying that Yap1 activation alone is inadequate to stimulate physiological growth. Consequently, the effects noted on cardiac hypertrophy may be facilitated by Yap1-independent mechanisms downstream of lncExACT1 and DCHS2 (Li et al., 2022).

A recent analysis of microRNAs indicated that the expression of miR-222, a protein vital to cardiac function, fluctuates in response to physical training, with its therapeutic cardioprotective properties upregulated in two exercise models and heightened in patients with heart failure (Liu et al., 2015). These properties were demonstrated in mice that overexpressed MiR-222, which prevented exercise-induced cardiac hypertrophy. There is a correlation between elevated levels of the cardiac renin-angiotensin system (RAS), which includes angiotensinogen, angiotensin-converting enzyme (ACE), angiotensin II, and left ventricular hypertrophy. Through the regulation of the equilibrium between these substances, ACE2, a newly discovered component of the RAS, contributes to the preservation of cardiovascular homeostasis. The levels of ACE and Ang II in the heart can be decreased through swimming exercises, while the levels of ACE2 and Ang can be increased (Iwai et al., 1995; Adamcova et al., 2021). Through aerobic exercise, miR-27a and -27b can be increased, which targets atrial fibrillation (ACE), and miR-143 can be reduced, which causes left ventricular hypertrophy. In rats, exercise-induced cardiac hypertrophy leads to a reduction in the expression of miR-29, along with downregulation of miR-133b, -1, and -133a, and upregulation of miR-29a. The expression of miR-29a is correlated with a lower concentration of hydroxyproline and collagen in the left ventricle (LV), which suggests that the LV is more compliant and has beneficial effects on the heart (Rodriguez et al., 2024). The expression of miR-145, -21, -144, is found to be upregulated in response to swimming exercise training-induced left ventricular remodeling, whereas the expression of miR-124 is found to be downregulated. It appears from this that activation of PI3K/AKT/mTOR is essential for the development of cardiac hypertrophy. It has been discovered that particular microRNAs are capable of targeting the PI3K/AKT/mTOR signaling pathway as well as its negative regulators (Da Silva et al., 2012). A rat model of cardiac hypertrophy induced by prolonged swimming was used to investigate the relationship between microRNAs and the PI3K/AKT/mTOR pathway. The results of this study showed that miR-19b, -133b, -30e, and -208a exhibited upregulation, whereas miR-191a, -99b, -22, -100, and -181a exhibited downregulation (Ramasamy et al., 2015). It has been established that the majority of microRNAs, such as miR-19, -208, -99, -100, and, -181, are associated with cardiac hypertrophy and apoptosis, primarily through the MAPK and the PI3K/Akt/mTOR signaling pathways. By gaining an understanding of the microRNAs and cellular pathways that regulate exercise-induced cardiac hypertrophy, it may be possible to accelerate the development of treatments for cardiovascular disease (Zhang et al., 2020). MiRNA-195 is upregulated during cardiac hypertrophy, resulting in pathological hypertrophy and heart failure in mice. It is integral to hypertrophic growth and cardiac remodeling resulting from pathological signaling (van Rooij et al., 2006). MiR-208, a cardiac-specific microRNA, is essential for hypertrophy, fibrosis, and the upregulation of βMHC in cardiomyocytes under stress conditions. MiR-133 and miR-1 are downregulated in models of cardiac hypertrophy, and their overexpression mitigates hypertrophy. Inhibition of MiR-133 results in considerable cardiac hypertrophy and dysfunction, indicating its cardioprotective role in hypertrophic conditions by upregulating the targets Rho1, Cdc42, and Nelf-A/WHSC2. MicroRNAs such as miR-1, miR-23a, and miR-34 can influence cardiac hypertrophy, either providing protection or exacerbating the condition, underscoring their significance as prospective therapeutic targets in the treatment of heart disease (Callis et al., 2009; Carè et al., 2007). In a study that investigated the expression of microRNAs in exercise-induced left ventricular hypertrophy, researchers discovered that after 35 days, the levels of miR-150 increased, while the levels of miR-143, -26b, and-27a, decreased after 7 days. To confirm the targets of these microRNAs and to gain a better understanding of their roles in the adaptation of the heart to physical training, additional research is required (Sanchis-Gomar et al., 2021).

MiRNA-541-5p may function as a biomarker for myocardial ischemia/reperfusion injury, modulating oxidative stress and ferroptosis through the inhibition of its expression (Zhao et al., 2025). The research indicates that miRNA-27a may intensify ferroptosis in brain tissue during ischemic stroke by potentially suppressing Nrf2. *In vitro* investigations demonstrated that miR-182-5p and miR-378a-3p promote ferroptosis in cells by modulating the expression of GPX4 and SLC7A11. *In vivo* studies demonstrated that silencing these genes mitigated ischemia/reperfusion-induced renal injury in rats (Zhang et al., 2022). MiR-126, Spred-1, and PI3KR2 inhibit angiogenesis in a rat swimming model by decreasing heart rate and blood pressure, while enhancing the expression of anti-angiogenic microRNA in the soleus muscle (Improta Caria et al., 2018). Physical activity promotes angiogenic factors in hypertension, inhibits apoptotic signaling, and modulates vascular disease. MicroRNAs induced by exercise mitigate hypertension-related microvascular abnormalities by preserving the equilibrium between angiogenic and apoptotic factors (Fernandes et al., 2012). Recent studies demonstrate that miR-221 can attenuate VEGF receptor signaling through the regulation of PI3K regulatory subunits. The MiR-221/222 family, a target gene of c-Kit and let-7f, facilitates angiogenesis via thrombospondin 1, as evidenced by deep sequencing in zebrafish embryos. Suppression of MiR-221 impairs embryonic vascular development, resulting in anomalies in angiogenesis and lymphatic vasculature, whereas overexpression promotes proliferation and migration in apical cell activity (Nicoli et al., 2012). The regulation of phosphoinositide-3-kinase by MiR-126 not only amplifies the effects of Ang-1 on vessel stabilization and maturation but also modulates VEGF signaling. It has been showed that miR-200b modulates angiogenic signals in endothelial cells by targeting VEGF signaling receptors, including VEGFR2, Flt1, KDR, and GATA binding protein 2. Another study indicated that miR-329 can suppress angiogenesis and arteriogenesis by targeting the co-receptor for VEGFR2, CD136, and MEF2a (Fish et al., 2008).

Research indicates that pathogenic cardiac stressors enhance miR-574 expression in humans and mice, resulting in cardiac dysfunction in knockout mice, whereas nanoparticle-mediated delivery alleviates this effect. Transcriptomic analysis of miR-574-deficient hearts revealed FAM210A as a common target mRNA of miR-574-5p and miR-574-3p. Modulating miR-574 deficiency or FAM210A expression influences the protein levels of mitochondrial-encoded electron transport chain genes, potentially impacting cardiac remodeling in heart failure (Wu et al., 2021). MicroRNA-574, particularly the 5p arm, is integral to mitochondrial biogenesis, modulating the expression of genes such as PGC-1α and SIRT1, which are vital for mitochondrial function (Wu et al., 2021). MicroRNA-21, a vital microRNA in the cardiovascular system, is indispensable for the proliferation of vascular smooth muscle cells, apoptosis, cardiac cell growth, and fibroblast activity. MiR-21, a gene associated with the pathogenesis of cardiovascular diseases, may represent a potential therapeutic target for Programmed Cell Death 4, PTEN, SPRY1, and SPRY2 (Cheng and Zhang, 2010).

Frank et al. have shown that long non-coding RNAs (lncRNAs) are essential in the advancement of cardiac hypertrophy (Frank et al., 2016). Researchers identified that CHAST, a transcript associated with cardiac hypertrophy, is upregulated in mice subjected to pressure overload, a condition capable of inducing cardiac hypertrophy. LncRNAs can engage with chromatin remodeling factors, modifying chromatin structure and establishing a feedback loop that governs lncRNA expression. The lncRNA Mhrt and chromatin feedback loop are essential for cardiac function and the prevention of pathological hypertrophy, whereas the restoration of Mhrt expression safeguards against heart failure (Viereck et al., 2016; Goh et al., 2019). A study examines the correlation between MALAT1 and miR-320a and the improvement of endothelial dysfunction in obese children and adolescents, emphasizing the potential protective benefits of exercise (Montero et al., 2012). Exercise training diminishes levels of VCAM-1, ICAM-1, and E-selectin in obese children and adolescents, suppresses MALAT1 expression, and elevates miR-320a expression, indicating that physical activity may safeguard against endothelial dysfunction (Zhao et al., 2021). The MALAT1/miR-320a axis may be associated with the beneficial effects of exercise on endothelial function in obese children and adolescents, as evidenced by their correlation with markers of endothelial dysfunction.

## 5 The diagnostic and clinical efficacy of microRNAs and lncRNAs in the cardiovascular diseases and heart failure

Recent studies indicate that microRNAs, functioning as biomarkers for health and disease, mature within the circulatory system and associate with RNA-binding proteins such as Argonaute2 or HDL/LDL (Gayosso-Gómez and Ortiz-Quintero, 2021). Extracellular vesicles are also responsible for this release. The contents of vesicles are being transported to target cells by ci-miRNAs. ci-miRNAs, which can be extracted from bodily fluids, demonstrate stability after freezing, thawing, and temperature fluctuations, which suggests that they could serve as biomarkers for monitoring these conditions. Exercise affects the expression of miRNA in tissue and circulation (Xu et al., 2022). A profile of specific ci-miRNAs associated with angiogenesis (miR-222,-328, -, -20a, -221, and-210), inflammation (miR-21 and -146a), skeletal and cardiac muscle contractility (miR-133a and miR-21), and hypoxia/ischemia adaptation (miR--146a,- 210, and-21) has been examined in healthy competitive athletes before, during, and after acute exhaustive exercise testing (Kilian et al., 2016). The goal of this study is to identify exercise-induced changes in the expression of small interfering microRNAs in humans. It has been demonstrated that after 90 days of aerobic exercise training, there is an increase in the levels of microRNAs miR-221, -222, , -20a, -146a, and --21, and in that individual’s plasma (Sullivan et al., 2022). They discovered that peak levels of miR-146a and miR-20a exhibited a positive correlation with peak oxygen consumption (VO2 max), which suggests that there is a viable approach to utilizing ci-miRNAs as biomarkers for physical fitness. After that, the involvement of muscle-specific microRNAs (miR-1, -499, --208a, -208b, -133b, and 133a) as well as muscle-related microRNAs (miR—214, and-181) in physical activity was investigated. After exercise, particularly after downhill training, the plasma concentrations of microRNAs miR-1, miR-133a, and miR-133b significantly increased (Baggish et al., 2011). This was especially true after the exercise. Short-term increases in miR-181b and miR-214 expression were observed. To evaluate muscle-enriched ci-miRNA alterations, additional research is required. Plasma concentrations of miR-133 b, -1, and -133a, significantly increased after exercise, particularly after downhill training. This was especially the case. During this brief period, miR-181b and -214 were enhanced. Assessment of muscle-enriched ci-miRNA alterations requires additional research to be conducted (Sieland et al., 2021). A subsequent study discovered that an increase in miR-149 levels occurred during physical exercise, while miR-146a and -221 showed significant reductions. The initial study discovered that a decrease in c-miR-486 can lead to metabolic changes during physical exercise. These changes may be influenced by the type of exercise, the duration of the exercise, and the intensity of the exercise, according to the findings of the study, which found that muscle-specific miRNA concentrations did not change after acute resistance exercise (Sawada et al., 2013). MiR-126, a biomarker for endothelial cell damage, was utilized in the study to analyze plasma samples obtained from healthy subjects following endurance exercise evaluations. These evaluations included maximal symptom-limited tests, cycling, marathon running, and resistance training. Endothelial damage is indicated by elevated levels of microRNAs miR-126 and -133, which are produced as a result of endurance exercise protocols (Uhlemann et al., 2014). Increasing the levels of miR-133 is another benefit of eccentric resistance training. As indicated by elevated biomarkers, the completion of a marathon causes significant damage to the skeletal muscles, stressful conditions for the cardiac muscles, and inflammation throughout the body. During the process of dynamic regulation of ci-miRNAs, a group of miRNAs participates in the process both before and after the completion of extended, submaximal aerobic training (for example, marathon running) (Siracusa et al., 2018). Ci-miRNAs associated with vascular endothelium (miR-126) and inflammatory miRNAs (miR-146a) are present at relatively elevated levels in plasma during resting conditions before marathon running. On the other hand, cardiac and muscle-enriched ci-miRNAs (miR-499–5p, -1, and -133a) exhibit significantly low expression in plasma during these conditions (Baggish et al., 1985). All candidate ci-miRNAs displayed a significant increase immediately after the marathon, and then within twenty-four hours after the race, they returned to the levels they had been at before the race or even decreased altogether. There was a significant increase in the levels of miR-499, -133a, -1, -208b, and -206 in humans before, immediately after, and 24 h after the marathon, according to a report that was published more recently on heart- and muscle-specific microRNAs (Ramos et al., 2018). While the levels of miR-499 and -208b returned to their initial levels, the levels of the other microRNAs remained elevated twenty-four hours later. According to the findings of the study, there were significant correlations between aerobic performance capacity, VO2 max, running speed, miR-206, -1, and -133a. On the other hand, the expression of miR-21 and miR-155, which are related to fibrosis and inflammation, was not affected by exercise (Mooren et al., 2014). Following acute endurance exercise and a 12-week endurance training regimen, a comprehensive ci-miRNA analysis was performed on young, healthy males. The results showed that the levels of ci-miRNA increased, particularly for miR-139-5p, −1, -338-3p, -330-3p, −223, and 143. On the other hand, eight ci-miRNAs, including let-7i, miR-221, -151-3p, -146a, -106a, -30b, -652, and -151-5p, showed a reduction after the exercise (Wang H. et al., 2017). In addition, chronic modifications of ci-miRNAs have been evaluated after undertaking an endurance training regimen for 12 weeks. Let-7d, miR-21, -148a, -25, -342-3p, miR-766, and miR-185, were among the seven ci-miRNAs that showed a significant decrease after the training period (Xu et al., 2015). On the other hand, miR-103 and -107 were among the two ci-miRNAs that showed a significant increase after the training period. One study investigated the global response of inflammation-related microRNAs (c-inflammamiRs) to various exercise doses. Moderate aerobic exercise has been linked to powerful anti-inflammatory mechanisms, and the study was conducted to investigate this relationship (de Gonzalo-Calvo et al., 1985). An increase in miR-150-5p was found immediately after the 10-kilometer race, while the levels of 12 c-inflammamiRs rose immediately after the marathon (miR-148a-3p, -143-3p, -424-5p, -223-5p, -125b-5p, -132-3p, -223-3p, -29a-3p, -34a-5p, -424-3p, let-7d-3p, and let-7f-2-3p). These findings were discovered from the profiles of c-inflammamiRs before, immediately after, and 24 h after participation in marathon and 10-kilometer races (Domańska-Senderowska et al., 2019; Abd El-Kader et al., 2013). According to the findings of the study, the levels of microRNA named miR-193b and -192 in humans with prediabetes and mice that are unable to tolerate glucose have the potential to act as a novel biomarker for prediabetes and a key indicator for therapeutic exercise intervention. According to the findings of the study, circulating microRNAs have the potential to act as a real-time biomarker that is non-invasive and can be used to investigate exercise-induced tissue adaptation (Ma et al., 2022).

Research is underway on cardiovascular non-coding RNA therapies for conditions such as cardiovascular hypertrophy, disrupted excitation–contraction coupling, cellular apoptosis, interstitial fibrosis, and microvascular rarefaction. A common approach to inhibit miRNAs implicated in cardiac hypertrophy involves the utilization of 2′-OMe-modified antagomiRs or locked nucleic acids (LNAs) (Jalink et al., 2024). Numerous miRNAs that function in either cardiomyocytes or fibroblasts have been shown to inhibit hypertrophy and remodeling during experimental heart failure in mice. Alternative cell-type-specific approaches may also prove effective, including the inhibition of leucocyte-expressed miR-155, angiogenesis, osteopontin, endothelial miR-24, and the targeting of lncRNA Chast (Topkara and Mann, 2010). Multiple ncRNA therapeutics are being developed to treat hyperlipidaemia, owing to the liver’s accessibility as a target. Lipoprotein A (LPA) is a crucial target that remains unaddressed by small-molecule therapies, yet it serves as a primary carrier of oxidised phospholipids in human plasma and is a causal risk factor for atherosclerotic cardiovascular disease and aortic stenosis. Pelacarsen, an ASO-targeting LPA mRNA, reduces Lp(a) levels by as much as 80% and is generally well tolerated, except for injection-site reactions (Thau et al., 2024). An antisense oligonucleotide directed at LPA mRNA is conjugated with triantennary N-acetylgalactosamine to facilitate the therapy. Olpasiran, a GalNAc-conjugated siRNA that reduces Lp(a), is presently undergoing Phase 2 trials (NCT04270760), while SLN360, another GalNAc-conjugated siRNA, is in Phase 1 trials (NCT04606602). Angiopoietin-like 3 (ANGPTL3) represents a compelling target for hyperlipidaemia, as studies indicate that loss-of-function variants are associated with markedly reduced LDL-cholesterol and triglyceride levels, along with a diminished risk of coronary heart disease (CHD) (Sosnowska et al., 2022). The circulating inhibitor of lipoprotein lipase and endothelial lipase, ANGPTL3, predominantly synthesized in the liver, regulates the absorption of muscle-free fatty acids, lipogenesis in adipose tissue, and the hepatic uptake of LDL and residual cholesterol. Inhibition of miR-92a using antisense oligonucleotides enhances wound healing, accelerates re-endothelialization, and prevents endothelial dysfunction and atherosclerosis in murine and porcine models following myocardial infarction and hind limb ischemia (Sylvers-Davie and Davies, 2021). In swine, the delivery of an anti-miR-92a LNA ASO through catheter markedly reduced infarct size and improved cardiac function. A LNA ASO aimed at miR-132-3p for heart failure patients has presented initial human data (Gallant‐Behm et al., 2018).

## 6 Conclusion and future prospects

Consistent physical activity directly influences skeletal muscle performance, nutrient metabolism, and muscular strength, while diminishing the risk of cardiovascular disease, type 2 diabetes, and specific cancers. MicroRNAs have facilitated a deeper comprehension of the molecular mechanisms regulating exercise-induced adaptations in muscles, cardiac tissue, and vascular systems. According to the findings of a large number of studies conducted on both animal models and human subjects, microRNAs are subject to dynamic regulation in response to physical activity. In contrast to the comprehensive research that has been conducted on miRNAs in the field of fitness, there is a lack of knowledge concerning lncRNAs in the field of exercise training. to gain a better understanding of the role that lncRNAs play in exercise adaptation in skeletal muscle, the heart, and the circulatory system, additional research and development are required. The identification of the exercise-induced signals that regulate microRNAs and lncRNAs will be essential for the development of therapy, and it will also determine whether or not physical activity is the most effective intervention in the prevention of disease development such as cardiovascular disease and heart failure. Interestingly, microRNAs and lncRNAs are integral to the advantageous effects of exercise on cardiac health and the progression of certain cardiovascular diseases. Investigating their response to exercise may yield novel pharmacological treatments for cardiovascular diseases or therapeutic approaches. Nonetheless, disparities between human and murine species must be acknowledged before the implementation of therapeutic strategies from the laboratory to clinical practice. Muscle-specific c-miRNAs are examined more thoroughly, whereas non-muscle-specific variants are investigated less frequently. Investigating exercise-responsive, non-muscle-specific circulating miRNAs is essential for comprehending their roles and potential application as biomarkers for physical performance. The investigation of circulating microRNAs in blood as prospective biomarkers is highly significant. C-miRNAs can be released into the bloodstream via association with lipid vesicles or proteins, facilitating intercellular communication. Lipid vesicles derived from various cells and tissues display distinct secretion properties and carrier selectivity upon release into the bloodstream. c-miRNAs can be taken up by remote tissue cells, modulating metabolism and intercellular communication. The degree to which c-miRNAs can be released from particular tissues and perform paracrine functions is yet to be explored. Additional investigation is required to comprehend their function in intercellular communication. On the other hand, recent results have proved that exercise causes significant reductions in blood pressure, as well as a reduced risk of subsequent cardiovascular incidents and mortality caused by cardiovascular disease. As of right now, a great number of lncRNAs that have been associated with cardiovascular diseases and regular exercise have been discovered through the use of RNA sequencing (RNA-seq) and bioinformatics analyses. In the context of cardiovascular diseases, the clinical relevance of lncRNAs is substantial, and they present a variety of opportunities for diagnosis and treatment opportunities. Furthermore, lncRNAs exhibit distinct expression patterns that are specific to a variety of tissues and cell types. This makes it easier to classify them into distinct subclasses of cardiovascular diseases and potentially predict how patients will react to treatment. On the other hand, our current understanding of the impact that lncRNAs have on cardiovascular diseases is probably limited. On account of this, it is of the utmost importance to carry out investigations that are more comprehensive to enhance our understanding of the mechanisms by which lncRNAs influence cardiovascular diseases and contribute to the development of novel therapeutic approaches.

Exercise training is a non-pharmacological intervention employed to prevent and manage CVD and heart failure. Nonetheless, evidence connecting epigenetic modifications to alterations in the heart and blood vessels induced by exercise is scarce. Current evidence indicates that ET’s protective effects entail alterations in the expression patterns of specific miRNAs and lncRNAs that positively influence cardiovascular remodeling. MicroRNAs (miRNAs) and lncRNAs are essential in regulating numerous physiological processes in mammals, such as cell proliferation, differentiation, migration, angiogenesis, apoptosis, tissue development, and remodelling. Further research is required to elucidate the pathways and mechanisms through which ET influences miRNAs in correlation to lncRNAs, and to expand the scope of ET’s application in rectifying pathological processes via miRNA and lncRNAs therapy. This may aid in the prevention of cardiovascular and heart damage, enhancing patient survival and quality of life.
